# A contemporary overview of severe community-acquired bacterial infections in pediatric intensive care units

**DOI:** 10.1016/j.aicoj.2026.100112

**Published:** 2026-07-09

**Authors:** Michael Levy, Annelise Mary, Fleur Cour Andlauer, Jeremie Rousseaux, Lionel Berthomieu, Camille Brehin, Stephane Bechet, Montserrat Sierra-Colomina, Camille Aupiais, Francois Angoulvant, Elise Launay, Damien Dubois, Robert Cohen, Stephane Leteurtre, CAPRICE Study Group, Julien Baleine, Laurent Balu, Simon Barreault, Clémence Beauruelle, Emma Becuwe Gosselin, Matthieu Bendavid, Xavier Beretta Piccoli, Lionel Berthomieu, Stéphane Blanot, Stephane Bonacorsi, Yvonnick Boué, Noel Boussard, Gérald Boussicault, Camille Brehin, Alexandre Bretonneau, Arthur Charazac, Camille Chavy, Maryline Chomton Cailliez, Benoit Colomb, Fleur Cour Andlauer, Julie Cremninter, Etienne Darieux, Stephane Dauger, Alexandra David, Sandrine De Sampaio, Damien Dubois, Audrey Dupont, Jean Duval Destin, Aben Essid, Marion Favre, Fiona Bensoussan, Ghida Ghostine, Lucie Gibold, Marie-Laure Girardin, Chloe Hild, Sylvia Iacobelli, Charlotte Idier, Christophe Isnard, Benoit Jaulhac, Katy Jeannot, Julien Jegard, Mikael Jokic, Aurélie Labarre, Elise Launay, Cécile Le Brun, Michael Levy, Alain Lozniewski, Annelise Mary, Fabrice Michel, Christophe Milesi, Julie Missaire, Guillaume Mortamet, Simon Mounier, Audrey Mowendabeka, Jérôme Naudin, Anouk Navion, Mehdi Oualha, Naim Ouldali, Bruno Ozanne, Carla Parada-Rodrigues, Manon Passard, Quentin Pledran, Amandine Prenant, Pauline Ragon, Jérôme Rambaud, Sophie Reissier, Chloé Ribet, Claire Richarme, Barbara Ros, Jean-Michel Roué, Nicolas Roullet-Renoleau, Jérémie Rousseaux, Montserrat Sierra Colomina, Laurence Tabone, Muhamed-Kheir Taha, Asmaa Tazi, Aurore Thollot, Nicolas Traversier, Anne Tristan, Lucile Tzaroukian, Noemie Vanel, Diane Vareilles, Emmanuelle Varon, Laurent Villeneuve, Evelyne Werner, Arnaud Wiedemann, Amani Zaouali Dridi, Justine Zini, Charlie Zins, Corinne Levy, Etienne Javouhey

**Affiliations:** a Pediatric Intensive Care Unit, Strasbourg University Hospital, University of Strasbourg, Strasbourg, France; b INSERM (French National Institute of Health and Medical Research), UMR 1260, Regenerative Nanomedicine (RNM), FMTS, Strasbourg, France; c French Group of Pediatric Intensive and Emergency Care (GFRUP), France; d French Group of Pediatric Infectious Diseases (GPIP), France; e Pediatric Intensive Care Unit, Robert Debré University Hospital, Assistance Publique-Hôpitaux de Paris, Université Paris Cité, Paris, France; f Pediatric Intensive Care Unit, Hôpital Femme Mère Enfant, Hospices Civils de Lyon, University Lyon 1, Lyon, France; g Pediatric Intensive Care Unit, Centre Hospitalier Universitaire Lille, Université de Lille, Lille, France; h Pediatric Intensive Care Unit, Toulouse University Hospital, University of Toulouse, Toulouse, France; i Department of Pediatrics, Toulouse University Hospital, University of Toulouse, Toulouse, France; j Association Clinique et Thérapeutique Infantile du Val-de-Marne (ACTIV), Créteil, France; k IMRB-GRC GEMINI, Centre Hospitalier Intercommunal, Research Centre, Université Paris Est, Créteil, France; l Pediatric Emergency Department, Jean Verdier Hospital, Assistance Publique-Hôpitaux de Paris, Université Paris Cité, Bondy, France; m Université Paris Cité, ECEVE, UMR 1123 Unit, Inserm, France; n Department of Paediatrics, Department Woman-Mother-Child, Lausanne University Hospital (Centre Hospitalier Universitaire Vaudois), Lausanne, Vaud, Switzerland; o Department of Pediatrics, Centre Hospitalier Universitaire Nantes, Université de Nantes, Nantes, France; p Nantes Université, Inserm UMR 1307, CNRS UMR 6075, France; q Bacteriology and Hygiene Laboratory, Toulouse University Hospital, University of Toulouse, Toulouse, France; r Univ.Lille, CHU Lille, ULR 2694 - METRICS: Évaluation des Technologies de Santé et des Pratiques Médicales, F-59000 Lille, France; s EA 7426, Pathophysiology of Injury-Induced Immunosuppression, University Claude Bernard Lyon 1, BioMérieux Hospices Civils de Lyon, E. Herriot Hospital, 69003, Lyon, France

**Keywords:** Bacteria, Epidemiology, Infection, Sepsis, Septic shock

## Abstract

**Background:**

Community-acquired bacterial infections (CABIs) remain a leading cause of pediatric morbidity and mortality. This study aimed to provide a contemporary description of pediatric CABIs requiring admission to pediatric intensive care units (PICUs) in France.

**Methods:**

The CAPRICE study is a prospective, multicenter cohort including all children with suspected CABIs who were admitted to 28 French PICUs from January to December 2024. Demographic, clinical, microbiologic, and outcome data were prospectively collected. The primary outcome was mortality at day 28 and secondary outcomes included sequelae at day 28, PICU length of stay, and total hospital stay. Independent predictors of mortality were identified by multivariable logistic regression.

**Results:**

Among 897 children, the median age was 1.9 years (IQR 0.2−8.8); 55% were male, and 30% had a chronic condition. The leading infections were lower respiratory tract infection (43%), meningitis (20%), and ear-nose-throat infections (13%). Septic shock occurred in 17% of cases. A pathogen was identified in 89% of cases: *Bordetella pertussis* (22%) and *Mycoplasma pneumoniae* (15%) were predominant, followed by *Streptococcus pneumoniae* (13%), and *Staphylococcus aureus* (11%). In all, 34% patients had viral co-infection and 70% required organ support. Overall mortality was 7%, increasing to 19% with septic shock. Independent predictors of death included multiple organ failure, meningitis, and *B. pertussis* infection. Sequelae occurred in 15% of survivors.

**Conclusions:**

Severe CABIs in French PICUs in 2024 were mainly caused by *B. pertussis* and *M. pneumoniae*, followed by *S. pneumoniae* and *S. aureus*. Mortality and morbidity of these infections remain substantial.

## Background

Community-acquired bacterial infections (CABIs) continue to represent a major global health concern, particularly among children < 5 years old, despite prevention strategies and antibiotic treatments [1]. These infections are associated with substantial morbidity and mortality, leading to extensive use of antimicrobial agents and advanced supportive therapies as well as frequently prolonged hospital stays [[2], [3], [4]]. Reported mortality for pediatric CABIs requiring pediatric intensive care unit (PICU) admission varies widely across settings ranging from 6 to 10 % in high income settings [5] and up to more than 30 % in low- and middle-income settings [2,6]. For clinicians in PICUs, rapid recognition and appropriate management of such infections are critical, yet the pathogens responsible and their resistance patterns are subject to continuous changes [7,8].

Over the past 3 decades, vaccination campaigns have profoundly reshaped the epidemiologic landscape of pediatric bacterial infections. The introduction of conjugate vaccines against *Haemophilus influenzae* (Hi) type b, *Neisseria meningitidis* (Nm) and *Streptococcus pneumoniae* (Sp) has greatly reduced the incidence of invasive disease due to these pathogens, but Nm and Sp were still the main pathogens involved in community-acquired sepsis in European PICUs from 2012 to 2016 [5]. Since then, there have been shifts in the distribution of circulating serotypes, the emergence of non-vaccine strains, and the relative increase in the prevalence of other community-acquired bacterial agents such as *Staphylococcus aureus* (SA) and group A *Streptococcus* (GAS) [9,10]. The COVID-19 pandemic profoundly reshaped the circulation of infectious agents, viral and bacterial, inducing in the post-pandemic period a high incidence of bacterial infections, probably due in part to the immune debt of populations induced by lockdowns [11,12]. These evolving trends underscore the need for updated epidemiologic data specific to the PICU setting, where disease severity, pathogen distribution, and therapeutic constraints differ from the general pediatric population.

The aim of this study was to provide a contemporary overview of CABIs among children admitted to PICUs, with particular attention to clinical presentation, microbiologic etiology, and patient outcomes.

## Methods

### Caprice study and study sites

The Caprice (CommunautAry Pediatric bacteRial Infection in intensive CarE unit) project is conducted by the French Group of Pediatric Intensive Care (GFRUP) in collaboration with the French Group of Pediatric Infectious Disease (GPIP). This large, prospective, multicenter observational study was designed to study the epidemiology of severe CABIs in France, to investigate pathogen virulence associated with these infections and the host inflammatory and immunologic response to life-threatening bacterial infections in children. In 2024, the Caprice network consisted of 28 French PICUs (82% of all French PICUs).

### Study population

From January 1, 2024 to December 31, 2024, we prospectively recruited all children aged 3 days to 18 years who were admitted to the participating French PICUs with suspected CABI. CABIs included lower respiratory tract infections (LRTIs) (including diffuse pneumonia, lobar pneumonia and pneumonia with pleural empyema); meningitis; cerebral empyema; ear-nose-throat (ENT) (like mastoiditis, ethmoiditis, peritonsillar abscesses, etc.), abdominal, urinary tract, soft-tissue, and osteoarticular infections; bloodstream infections without a source; and other infections with suspected bacterial origin. The community-onset nature of the infection was defined by symptoms occurring at home or within the first 48 h of hospitalization [13]; the diagnosis was at the discretion of the attending physician according to clinical, biologic and radiologic criteria. One patient could have more than one clinical syndrome. We excluded patients with early-onset neonatal sepsis occurring during the first 72 h of life [14], healthcare-associated infections, related to medical devices such as central lines or occurring after 48 h of hospitalization [15], febrile aplasia, fungal infections and patients with parental refusal of data use.

### Ethical approval

No written consent was required for this study, but parents received oral and written information regarding the study, and all data were analyzed unless parents told the pediatrician that they refused to participate. This study was approved by the Créteil hospital Ethics Committee (IRB no. 2023-12-16, December 8, 2023) and was registered at ClinicalTrials.gov (NCT06260345).

### Data collection

Prospective data collected included demographic characteristics, comorbidities, clinical presentation, illness severity, management, microbiology, and outcomes. Patients were stratified by age group: < 3 months, 3–11 months, 12 months to < 5 years, 5 to < 12 years and 12–18 years. Chronic conditions were classified according to the pediatric complex chronic conditions classification system V3 [16]. Severity was assessed with the Pediatric Index of Mortality 3 (PIM3) [17], and organ failure was defined according to the PODIUM conference [18]. Sepsis and septic shock were defined according to the International Consensus Criteria for Pediatric Sepsis and Septic Shock [19] and the first patients included had a retrospective review to ensure consistency with this new definition released in February 2024. Identification of bacteria was by culture or nucleic acid amplification test of specific pathogens in sterile sites or non-sterile sites (Supplemental Table 1) if the clinical presentation was compatible (blood, cerebrospinal fluid, urine, pulmonary samples, pleural samples, nasal/throat swabs [for *Bordetella pertussis* and *Mycoplasma pneumoniae*], joint or abscess aspirates, and intraoperative fluid). The pathogens recorded included *B. pertussis, Escherichia coli, Group B streptococcus, GAS, Hi, M. pneumoniae, Nm, SA, Sp, oral streptococcus, Klebsiella, Fusobacterium, Salmonella* and others. Pathogen strains were isolated according to standard methods of each laboratory and sent to the national reference centers for serotyping when appropriate. The antibiotic susceptibility of pathogens was specified, and multidrug-resistant bacteria were defined according to international definitions [20]. Viral coinfections were recorded and diagnosed by nucleic acid amplification test, including respiratory, neurologic and digestive multiplex panels.

### Outcomes

The primary outcome was in-hospital mortality, assessed at day 28 after admission. Secondary outcomes included sequelae at day 28, PICU length of stay, and total hospital stay. Sequelae were defined as new impairments that were not present before PICU admission and were assessed at Day 28 by the treating physician. Reported sequelae included neurological, respiratory, renal, cutaneous, cardiovascular, and orthopaedic complications. Data were entered into standardized Web-based case report forms, with regular audits, teleconferences, and meetings ensuring consistency across PICUs.

### Statistical analysis

Categorical variables are expressed as number (percentage) and were compared by chi-squared or Fisher exact test. *P* < 0.05 was considered statistically significant. Continuous variables are expressed as median (interquartile range [IQR]). Logistic regression was used to identify independent associated factors. Variables with *p* <  0.20 on univariate analysis were included in multivariable models, and only those with *p* <  0.05 were kept in the final model. All analyses were conducted with Stata 18.1 (StataCorp, College Station, TX, USA).

## Results

**Fig. 1:**
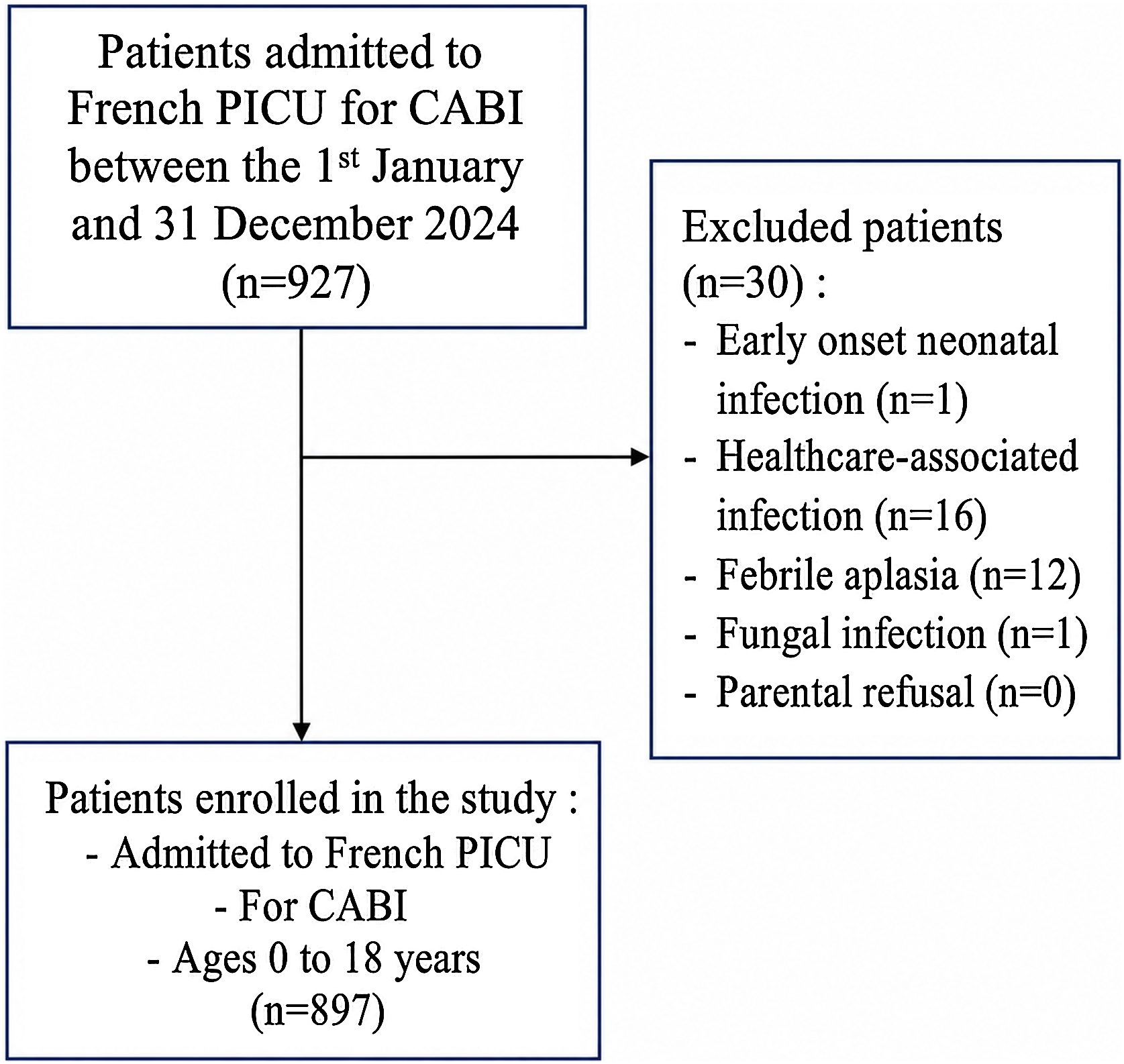
Patients admitted with a community-acquired bacterial infection to a French paediatric intensive care unit in 2024 (*N* = 897).

From January 1, 2024, to December 31, 2024, a total of 897 children with suspected severe CABIs were admitted to the 28 participating French PICUs (Fig. 1): 55% were male, and the median age was 1.9 years (IQR 0.2–8.8).

The main age group was < 3 months (28% of cases) (Table 1). Overall, 265 patients (30%) had an underlying chronic condition, most commonly neuromuscular disease (18%), respiratory disease (13%) and prematurity and complications of premature birth (13%). Only 30 patients (3.3%) had a known immune deficit. The proportions of chronic conditions increased in the different age groups reaching 41% in the 12−18 years age group (eTable 2).

**Table 1:** Characteristics of children with a community-acquired bacterial infection admitted to a French pediatric intensive care unit in 2024 (*N* = 897).

	Median (IQR), *n* (%)^a^
**Age, years**	1.9 (0.2–8.8)
<3 months	252/897 (28%)
3–11 months	134/897 (15%)
12 months - 5 years	176/897 (19%)
5–12 years	194/897 (22%)
12–18 years	141/897 (16%)
**Male sex**	470/852 (55%)
**Chronic condition**	265/874 (30%)
Neuromuscular	47/265 (18%)
Respiratory	36/265 (13%)
Prematurity and complications of premature birth	36/265 (13%)
Known immune deficit	30/265 (11%)
Hematologic	19/265 (7%)
Renal	17/265 (6%)
Cardiovascular	16/265 (6%)
Polymalformative syndrome	15/265 (6%)
Down syndrome without cardiopathy	12/265 (4%)
Other	37/265 (14%)
**Clinical presentation**	
Clinical syndrome^b^	
Lower respiratory tract infections	386/886 (43%)
Diffuse pneumonia	200/886 (22%)
Pleuropneumonia	102/886 (12%)
Lobar pneumonia	84/886 (9%)
Meningitis	175/886 (20%)
Ear-nose-throat infection	118/886 (13%)
Abdominal infection	46/886 (5%)
Cerebral empyema	46/886 (5%)
Urinary tract infection	43/886 (5%)
Soft-tissue infection	36/886 (4%)
Infection without a source	33/886 (4%)
Osteoarticular infection	14/886 (2%)
Other infection	104/886 (12%)
Sepsis or septic shock	
Sepsis^c^	114/881 (13%)
Septic shock^c^	149/881 (17%)
Toxic shock syndrome	85/884 (9%)
**Delay 1^st^ symptom to PICU admission, days**	4 (1–7)
Diffuse pneumonia	5 (2–8)
Pleuropneumonia	6 (3–9.5)
Lobar pneumonia	4 (2–7)
Meningitis	2 (1–3)
Ear-nose-throat infections	4 (2–7)
Abdominal infections	2 (1–5)
Cerebral empyema	5 (3–11)
Urinary tract infections	1 (1–3)
Soft-tissue infections	4 (2–8)
Infection without a source	1 (1–2)
Osteoarticular infections	5 (4–7)
Other infections	2 (1–7)
**Organ failure**	
Mortality PIM3 (%)	1.5 (0.9–3.3)
Organ failure^d^	505/856 (59%)
- respiratory	352/856 (41%)
*including ARDS*	69/856 (8%)
- cardiovascular	223/856 (26%)
- neurologic	156/856 (18%)
- renal	97/856 (11%)
- hepatic	54/856 (5%)
- gastrointestinal	30/856 (4%)
- hematologic	122/856 (14%)
- coagulation	72/856 (8%)
- immunologic	17/856 (2%)
- endocrinologic	13/856 (1%)

IQR: interquartile range; ARDS: acute respiratory distress syndrome; PICU: pediatric intensive care unit; PIM3: Paediatric Index of Mortality 3.

a Missing data: age = 0, sex = 45, chronic condition = 23, delay 1st symptom to PICU admission=15, clinical syndrome = 0, sepsis/septic shock = 16, PIM3 = 100, organ failure = 41.

b Patients could have more than one clinical syndrome.

c According to the Phoenix sepsis score.

d According to the PODIUM Consensus Conference.

### Clinical presentation

The median time from first symptoms to PICU admission was 4 days (IQR 1–7). The infections with the shortest median time between symptom onset and PICU admission were urinary tract infection, infection without an identified source, and meningitis (Table 1). The three main clinical syndromes were LRTI (43%), meningitis (20%) and severe ENT infections (13%). The proportion of clinical presentation according to age groups is displayed in eTable 2. The median PIM3 score of all patients with CABI was 1.5% (IQR 0.9–3.3). The four main organ failures were respiratory (41%), cardiovascular (26%), neurologic (18%) and hematologic (14%) failure (Table 1).

Among children admitted to a PICU for CABI, 114 (13%) had sepsis and 149 (17%) had septic shock. Patient with sepsis or septic shock were significantly older than patients without septic or septic shock (2.5 (IQR 0.4–10.7) vs 1.7 (IQR 0.2−8.1), *p* = 0.011) (eTable 3). The highest proportion of sepsis was observed in children aged 3–11 months, whereas the highest proportion of septic shock was observed in those aged 12–18 years. Patients with sepsis and septic shock had a shorter delay between 1st symptom and PICU admission compared with other patients (2 (IQR 1–4) days versus 4 (IQR 2−7) days, *p* = 0.005) (eTable 3). Meningitis, soft-tissue infections, abdominal infections and infections without a source were significantly associated with the presence of sepsis and septic shock. On the other hand, diffuse pneumonia was less frequently associated with sepsis or sepsis shock.

### Pathogens

Bacterial identification was obtained in 89% (794/897) of cases. The two main pathogens identified were *B. pertussis* (*n* = 177, 22%) and *M. pneumoniae* (*n* = 119, 15%) (Table 2). The other most frequent pathogens were Sp (*n* = 101, 13%) and SA (*n* = 85, 11%).

**Table 2:** Microbiology findings in children with a community-acquired bacterial infection admitted to a pediatric intensive care unit.

Main pathogens	*n* (%)^a^
*Bordetella pertussis*	177/794 (22%)
*Mycoplasma pneumoniae*	119/794 (15%)
*Streptococcus pneumoniae*	101/794 (13%)
PCV7 serotypes	4/54 (7%)
PCV13 serotypes	5/54 (9%)
PCV20 serotypes	10/54 (19%)
Non-vaccinal serotypes	35/54 (65%)
Non-determined	47
*Staphylococcus aureus*	85/794 (11%)
MRSA	13/85 (15%)
PVL	12/85 (14%)
*Escherichia coli*	62/794 (8%)
*Group A streptococcus*	61/794 (8%)
*Neisseria meningitidis*	53/794 (7%)
Group B	26/47 (55%)
Group W	13/47 (28%)
Group Y	8/47 (17%)
ND	6
*Haemophilus influenzae*	53/794 (7%)
Hi a	2/20 (10%)
Hi b	14/20 (70%)
Hi f	1/20 (5%)
NT Hi	3/20 (15%)
ND	33
*Group B streptococcus*	28/794 (4%)
*Oral streptococcus*	29/794 (4%)
*Salmonella*	13/794 (2%)
*Fusobacterium necrophorum*	14/794 (1%)
*Klebsiella*	11/794 (1%)
*Other*	57 (7%)
**Multidrug-resistant bacteria**	41 (5%)
Gram-negative	28/41 (68%)
MRSA	13/41 (32%)
**Viral co-infection**^b^	303/897 (34%)
Human rhinovirus/enterovirus	163/303 (54%)
Parainfluenzae	40/303 (13%)
Metapneumovirus	32/303 (11%)
Influenzae	29/303 (10%)
Respiratory syncytial virus	28/303 (10%)
Adenovirus	26/303 (9%)

MRSA: methicillin-resistant *Staphylococcus aureus*; PVL: Panton-Valentine Leucocidine-producing strain; MDR: multidrug-resistance; ND: not determined; NT: non-typable.

a A bacteria with identified in 794/897 (89%) of the included patients.

b Viral coinfection was explored only in 642 patients.

**Fig. 2:**
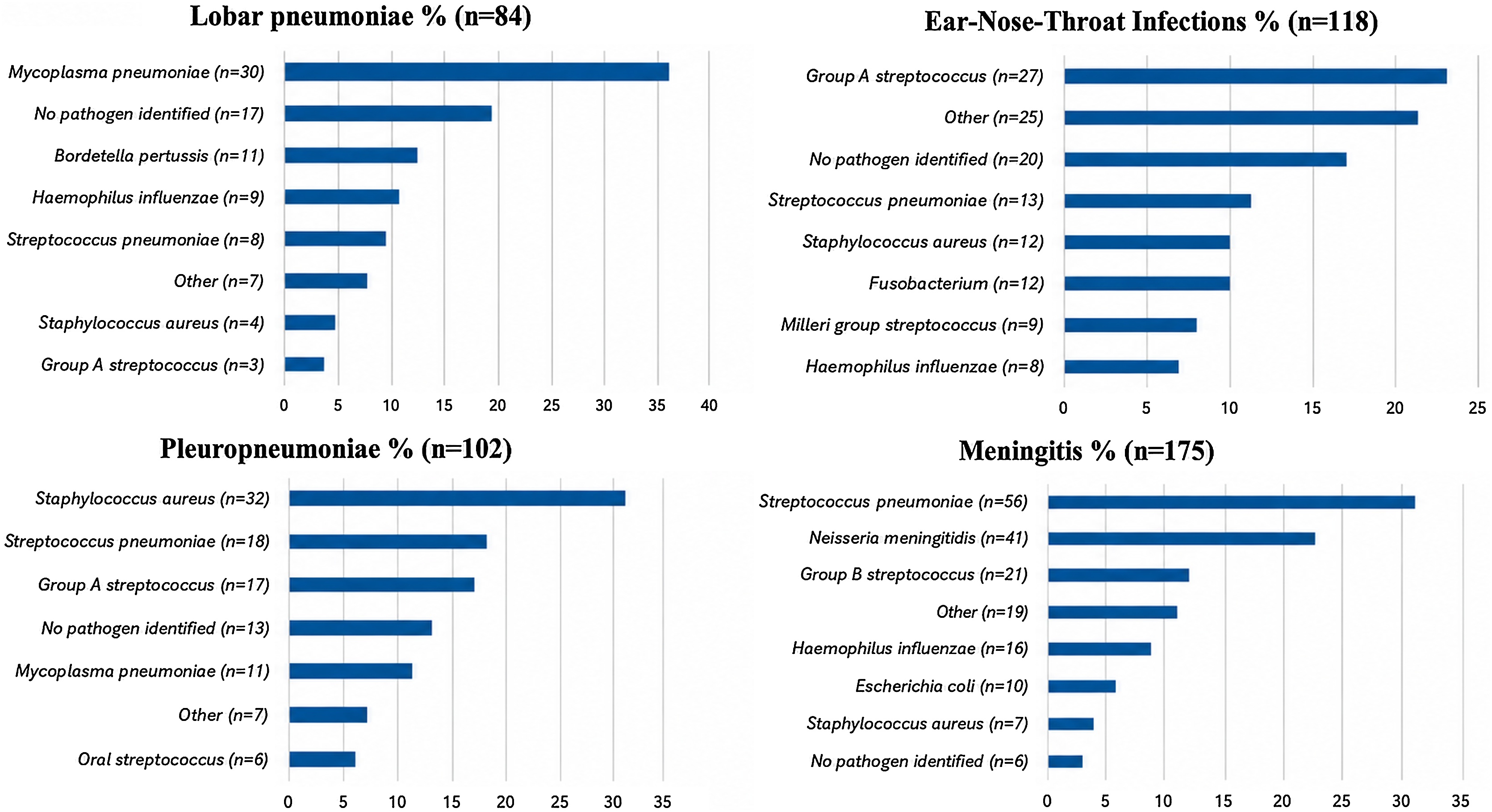
Invasive pathogens by site of infection (pulmonary, ear-nose-throat and meningitis) in patients with a community-acquired bacterial infection admitted to a French pediatric intensive care unit in 2024.

The pathogens involved in lobar pneumoniae, pneumoniae with pleural empyema, meningitis and ENT infections are in Fig. 2.

The three most frequent pathogens involved in septic shock were SA (25/149, 17%), Nm (23/149, 15%) and Sp (19/149, 13%). The two pathogens associated with an increased risk of sepsis or septic shock in case of CABI were Nm and SA (eTable 3).

The age distribution for each bacterial pathogen is in Table 3 and the temporal distribution of each pathogen during year 2024 is displayed in Supplemental Table 1.

**Table 3:** Invasive pathogens by age category in patients with a community-acquired bacterial infection admitted to a pediatric intensive care unit in 2024.

Pathogen	Total	Age group, n (%)				
		<3 months	3–11 months	12 months–5 years	5–12 years	12–18 years
*Bordetella pertussis, n (%)*	177	144 (81%)	22 (13%)	7 (4%)	4 (2%)	0 (0%)
*Mycoplasma pneumoniae, n (%)*	119	1 (1%)	6 (5%)	27 (23%)	51 (43%)	34 (28%)
*Streptococcus pneumoniae, n (%)*	101	7 (7%)	28 (28%)	28 (28%)	27 (27%)	11 (10%)
*Staphylococcus aureus, n (%)*	85	26 (31%)	23 (28%)	11 (13%)	10 (12%)	15 (18%)
*Escherichia coli, n (%)*	62	23 (37%)	11 (17%)	10 (16%)	9 (15%)	9 (15%)
*Groupe A streptococcus, n (%)*	61	3 (5%)	4 (7%)	21 (34%)	23 (38%)	10 (16%)
*Neisseria meningitidis, n (%)*	53	5 (9%)	9 (17%)	17 (32%)	14 (26%)	8 (16%)
*Haemophilus influenzae, n (%)*	53	4 (7%)	20 (38%)	13 (25%)	12 (23%)	4 (7%)
*Groupe B streptococcus, n (%)*	28	21 (75%)	5 (17%)	1 (4%)	1 (4%)	0 (0%)

Viral co-infection was explored in 72% of cases (*n* = 642) and identified in 34% (*n* = 303); the main virus was *Human rhinovirus/enterovirus* (Table 2).

### Therapy

Organ support was required for 70% of patients (Table 4). Invasive mechanical ventilation was required in 223 patients (26%), fluid resuscitation in 266 (27%), vasopressors in 166 (19%) and inotropes in 72 (8%). Corticosteroids were used in 22% of patients. They were used in 88/175 (50%) of patients with meningitis, 15/84 (18%) of patients with lobar pneumoniae and 59/149 (40%) of patients with septic shock.

**Table 4:** Treatments and outcomes of patients with a community-acquired bacterial infection admitted to a pediatric intensive care unit (*N* = 897).

	Median (IQR), *n* (%)
**Organ support**	625 (70%)
High-flow nasal oxygen	270 (30%)
Non-invasive ventilation	191 (21%)
Invasive ventilation	223 (26%)
Fluid ressuscitation	266 (27%)
Vasopressors	166 (19%)
Inotropes	72 (8%)
Corticosteroids	193 (22%)
Renal replacement therapy	29 (3%)
ECMO	22 (2%)
**Mortality at day 28**	
Overall mortality	60 (7%)
Mortality if sepsis or septic shock	34 (13%)
Mortality if septic shock	28 (19%)
Delay admission – death, days	4.5 (1–12)
**Length of stay, days**^a^	
PICU	5 (2–9)
Hospital	11 (6–19)
**Sequelae at day 28**	126/837 (15%)
Neurologic	69
Respiratory	17
Renal	15
Cutaneous	23
Cardiovascular	14
Orthopaedic	2

IQR: interquartile range; PICU: paediatric intensive care unit; ECMO: extracorporeal membrane oxygenation.

a Missing data: length of PICU stay = 30, length of hospital stay = 400.

### Outcomes

The overall mortality was 7% (*n* = 60) and increased to 19% (*n* = 28) for children with septic shock. On univariate analysis, risk of death was associated with the age groups 12 months to 5 years and 5–12 years, meningitis, septic shock and ≥ 2 organ failures (Table 5).

**Table 5:** Predictors of death in children with a community-acquired bacterial infection admitted to a pediatric intensive care unit: univariate and multivariate analysis.

	Survivors (*n* = 837)	Deaths (*n* = 60)	OR for death (95% CI)	*p*
**Univariate analysis**				
**Sex**				
Female sex	352/797 (44%)	31/55 (56%)	1.63 (0.94 ; 2.83)	0.081
**Age group**				
<3 months	222/837 (27%)	27/60 (45%)	Reference	
3-11 months	124/837 (15%)	8/60 (13%)	0.53 (0.233 ; 1.20)	0.129
12 months-5 years	165/837 (20%)	9/60 (15%)	0.44 (0.21 ; 0.98)	**0.044**
5-12 years	185/837 (22%)	8/60 (13%)	0.36 (0.16 ; 0.80)	**0.013**
12-18 years	130/837 (16%)	8/60 (13%)	0.51 (0.22 ; 1.15)	0.103
**Comorbidity**	241/816 (30%)	19/58 (33%)	1.16 (0.66 ; 2.05)	0.604
**Immune deficit**	27/809 (3%)	3/59 (5%)	1.55 (0.46 ; 5.27)	0.482
**Clinical presentation**				
Diffuse pneumonia	183/826 (22%)	16/60 527%)	1.28 (0.70 ; 2.32)	0.420
Pleuropneumonia	97/826 (12%)	4/60 (7%)	0.54 (0.19 ; 1.51)	0.239
Lobar pneumonia	78/826 (9%)	4/60 (7%)	0.68 (0.24 ; 1.94)	0.476
Meningitis	150/826 (18%)	23/60 (38%)	2.80 (1.62 ; 4.85)	**<0.001**
Ear-nose-throat infection	108/826 (13%)	5/60 (8%)	0.60 (0.24 ; 1.54)	0.292
Abdominal infection	44/826 (5%)	2/60 (3%)	0.61 (0.15; 2.59)	0.506
Cerebral empyema	41/826 (5%)	4/60 (7%)	1.37 (0.47 ; 3.95)	0.563
Urinary tract infection	40/826 (5%)	3/60 (5%)	1.03 (0.31 ; 3.45)	0.05
Soft-tissue infection	35/826 (4%)	1/60 (2%)	0.38 (0.05 ; 2.85)	0.348
Osteoarticular infection	11/826 (1%)	1/60 (2%)	1.26 (0.16 ; 9.89)	0.829
**Sepsis or septic shock**				
Sepsis	106/822 (13%)	6/59 (10%)	1.35 (0.54 ; 3.36)	0.64
Septic shock	121/822 (15%)	28/59 (48%)	5.51 (3.10 ; 9.77)	**<0.001**
**Pathogens**				
*Streptococcus pneumoniae*	91/826 (11%)	9/60 (15%)	1.46 (0.70 ; 3.07)	0.316
*Neisseria meningitidis*	48/826 (6%)	5/60 (8%)	1.47 (0.56 ;3.85)	0.429
Group A s*treptococcus*	58/826 (7%)	3/60 (5%)	0.70 (0.21 ; 2.29)	0.552
*Staphylococcus aureus*	79/826 (10%)	6/60 (10%)	1.05 (0.44 ; 2.52)	0.91
*Bordetella pertussis*	161/826 (19%)	16/60 (27%)	1.53 (0.84 ; 2.77)	0.166
Group B *streptococcus*	25/826 (3%)	3/60 (5%)	1.69 (0.49 ; 5.75)	0.404
*Haemophilus influenzae*	52/826 (6%)	1/60 (2%)	0.26 (0.03 ; 1.90)	0.183
*Escherichia coli*	59/826 (7%)	3/60 (5%)	0.68 (0.21 ; 2.25)	0.532
*Mycoplasma pneumoniae*	117/826 (14%)	2/60 (3%)	0.21 (0.05 ; 0.88)	0.032
**Number of organ failures**				
≥1	221/798 (28%)	5/58 (9%)	4.00 (0.77 ; 20.82)	0.099
≥2	228/798 (29%)	51/58 (88%)	40.48 (9.76 ; 167.92)	**<0.001**

OR: odds ratio; 95% CI: 95% confidence interval.

On multivariate analysis, risk of death was associated with ≥ 2 organ failures, meningitis and *B. pertussis* infection (Table 6).

**Table 6:** Multivariate predictors of death in children with community-acquired bacterial infection.

	Odds ratio	*p*	IC 95%	
Organ failure >=2	51.3	<0.001	11.6	226.6
Meningitis	4.5	<0.001	2.2	9.1
Pertussis	4.7	<0.001	2.2	10.4

Median PICU length of stay was 5 days (IQR 2–9) and median of hospital stay was 11 days (6–19).

At day 28, 126/837 (15%) patients had sequelae, mainly neurologic (Table 4).

## Discussion

This large multicenter prospective study provides a contemporary overview of CABI in critically ill children admitted to PICUs across France. This PICU network represent 82% of all PICU wards in France: the overall population of the country is 69,082,000 and the mean annual births over the last 10 years was about 700,000. With nearly 900 patients recruited over 1 year in this study, our findings illustrate both the latest microbiologic landscape of severe pediatric infections and the persistent burden of morbidity and mortality, despite improvements in vaccination coverage, antimicrobial therapy, and intensive care management. In fact, our findings highlight that severe CABIs are still responsible for consequent morbi-mortality. Overall mortality in our cohort was 7%, increasing to nearly 20% in children with septic shock, which is consistent with prior PICU-based sepsis studies [2,21]. Thus, despite advances in critical care, early recognition and timely intervention, severe cases still carry a high risk of death. On multivariable analysis, risk of death was associated with the presence of ≥ 2 organ failures, meningitis, and *B. pertussis* infection. These findings reinforce the importance of early recognition of infection with organ failure, including meningitis, for which prompt recognition is linked to better survival [22,23] They also reinforce the importance of prevention in severe pertussis in infants, in whom fulminant disease progression is well documented and survival poor even in cases of extracorporeal membrane oxygenation [24,25]. In addition, in our cohort, 15% of survivors had sequelae at day 28, mainly neurologic. This proportion is notably lower than in the EUCLIDS study [5], a large prospective multicenter cohort of 795 children admitted with community-acquired sepsis to 52 European PICUs, in which 31% of survivors were discharged with disability, including 24% of previously healthy children. It is also lower than the range reported in a scoping review of functional outcomes in pediatric sepsis survivors, where overall disability at hospital discharge or by 28 days ranged from 23% to 50% across studies [26]. Beyond neurologic impairment, both comparators capture broader functional domains — respiratory, motor, feeding, and cognitive — consistent with other pediatric sepsis cohorts where physical sequelae and ICU-acquired weakness contribute substantially to overall morbidity alongside neurologic outcomes. The lower rate observed in our cohort may reflect differences in case mix, in disability definitions and ascertainment methods, or in the severity profile of admitted patients, factors that vary considerably across studies and limit direct comparability.

The significant morbi-mortality concern all age groups including infants < 3 months old, who represented the largest age group admitted. This age-related vulnerability has been consistently reported in previous pediatric sepsis cohorts and likely reflects immature host defenses, lack of immunization by vaccines (including *B. pertussis*, Sp and Nm) as well as probable delayed recognition of infection in this population [27,28]. In addition, 70% of cases occurred in children without any underlying condition, which emphasizes that host susceptibility alone does not account for the severity of these infections even if these infections might reveal an immune deficit [29]. This finding is consistent with previous studies of severe pediatric infections. In the EUCLIDS study [5], 36% of patients had an underlying condition, while Lorton et al. reported comorbidities in only 24.5% of children with severe CABI [9]. These findings also suggest that severe bacterial infections and sepsis frequently occur in previously healthy children. Although chronic diseases are recognized risk factors for invasive bacterial infections and critical illness, they were not independently associated with mortality in our study. This may reflect the predominant influence of acute disease severity, organ dysfunction, and septic shock on outcomes once critical illness has developed.

Regarding epidemiology, the spectrum of pathogens we identified confirms the evolving epidemiology of pediatric bacterial infections following widespread implementation of conjugate vaccines targeting Hi type b, Nm, and Sp. In France in 2024, 81.9% of infants had received a first dose of the Nm B vaccine and 88.6% had received a first dose of the Nm C vaccine. Regarding Sp in 2022, 95.7% of infants aged 24 months had received the recommended vaccine schedule (2 + 1 doses) [30,31]. Nevertheless, our data demonstrate that Sp and Nm remain important causes of meningitis and septic shock. This finding agrees with previous reports in European PICUs [5], and the persistence of these infections likely reflects serotype replacement and incomplete vaccine coverage, which emphasizes the need for continuous epidemiologic surveillance and updated vaccine formulations. Furthermore, a major finding of our study was the unusual predominance of *B. pertussis* and *M. pneumoniae* as causative pathogens for severe CABIs in 2024. The predominance of *B. pertussis* agrees with many observations worldwide with an important peak in 2024 [[32], [33], [34]] and is explained by the cycling epidemiology of the disease, with repeated peaks not sustained in subsequent years, together with reduced population immunity after COVID-19 measures limited natural exposures, modifying the expression of virulence factors (pertactin and FIM2 gene) in circulating bacterial strains and possibly the detection of macrolide-resistant isolates [35]. This situation could also explain the high burden of CABIs in infants < 3 months old in our study. Although maternal immunization against pertussis was recommended in France in 2022, in 2024, the coverage was low, < 10%. Thus, these concerns once again reinforce the importance of maternal vaccination strategies. In addition, many countries experienced a strong outbreak of *M. pneumoniae* infection after the COVID-19 pandemic, with an increasing burden reaching high rates in 2024 [32,36,37]. Of note, *M. pneumoniae* was also responsible for lobar pneumoniae in our pediatric study, also described in adults in up to 40% of cases [36]. Apart from these two pathogens, Sp remained the first bacterium involved, together with SA [38]. Furthermore, in pleural empyema, which increased in incidence in many countries after the COVID-19 pandemic [39], the prominence of SA and GAS may reflect both the success of conjugate vaccines against encapsulated bacteria and the natural epidemiologic replacement by organisms not targeted by current vaccines, as previously reported. These findings highlight the need for vigilance regarding toxin-mediated disease and the potential benefit of adding anti-toxin antibiotics [40]. Finally, the frequent detection of viral co-infections in up to one third of tested cases, particularly *human rhinovirus-enterovirus*, raises important questions about pathogen interactions and the contribution of viral priming to bacterial disease severity [16]. Hence, vaccination against viruses could be useful to prevent CABIs. In fact, influenza vaccine programs are efficient in preventing severe bacterial super-infection or co-infection with increased related mortality [41]. Likewise, respiratory syncytial virus vaccination has been shown to reduce the incidence of acute otitis media in children [42], and respiratory syncytial virus and Sp infections were found closely linked [43].

LRTI was the most frequent clinical presentation, together with meningitis, which is still consistent with prior reports [2,5,21]. The high proportion of respiratory and cardiovascular failures we observed, that not only included sepsis and septic shock, illustrates the severity of CABIs. The finding that one-third of children required invasive mechanical ventilation and nearly one-fifth required vasoactive support highlights the critical nature of these infections and the heavy burden placed on intensive care resources, although disparities exist depending on the setting [44].

The strengths of this study include its prospective design, large sample size, and inclusion of almost all French PICUs, thus ensuring a representative national picture. The standardized data collection, with centralized case reporting and quality checks, also enhanced the robustness of our results. Furthermore, with extensive microbiologic testing in the different centers, a pathogen could be identified in 89% of cases, which is a high rate as compared with previously published data [5,21].

However, the first limitation is that the study covered a single calendar year, so temporal trends and seasonal variations should be interpreted cautiously. Second, the study was conducted in a high-income country with high vaccination coverage, and results are probably not generalizable to low-resource settings. In addition, vaccination data were missing for a substantial proportion of patients across participating centers, and we could note evaluate the relationship with the epidemiology of CABI in our cohort. Third, although we believe that recruitment was close to exhaustive within participating centers because of the prospective design, the active involvement of local investigators, and the regular monitoring procedures implemented throughout the study, no external validation using the national PMSI database was performed to ensure exhaustivity. Finally, while mortality was a reliable endpoint, sequelae at day 28 were assessed by the treating physician without a standardized evaluation scale, limiting the interpretability and generalizability of these particular results.

## Conclusions

This study provides a contemporary snapshot of severe CABIs in French PICUs with a predominance of LRTIs, meningitis and ENT infections. Despite a persistent predominance of Sp, the epidemiology has shifted as compared with historical cohorts, with a high burden of SA infections and an extraordinary prevalence of *B. pertussis* and *M. pneumoniae* that emerged as leading pathogens in 2024. Mortality remained substantial (7% increased to 19% in case of septic shock), particularly associated with organ failures, meningitis and pertussis. These findings underscore the persistent burden of CABIs in children and thus a need for vigilance, early recognition, adequate empiric therapy, and continued investment in prevention strategies and pathogen surveillance in the PICU setting.

## CRediT authorship contribution statement

ML and EJ take responsibility for the content of the manuscript, including the integrity of the data and the accuracy of the data analysis. ML, LB, CB, CA, FA, EL, DD, CL and EJ conceptualized the study. ML led the literature review. ML, ALM, FCA, JR, LB, CB, MSC and all the members of the CAPRICE Study Group led the data curation. ML, SB and CL led the data analysis and visualization. ML and SB led the data interpretation. ML and EJ drafted the manuscript. All authors commented on the draft report. All authors contributed to critical revision of the manuscript for important intellectual content. All authors had full access to all the aggregated data in the study. All authors read and approved the final draft of the manuscript and had final responsibility for the decision to submit for publication.

## Funding

This work was supported by AXA assurances and ACTIV (Association Clinique Thérapeutique Infantile du Val de Marne, France). The study sponsors had no role in the design or conduct of the study; collection, management, analysis, or interpretation of the data; preparation, review, or approval of the manuscript; or the decision to submit the manuscript for publication.

## Data sharing statement

Anonymized data are available on reasonable request to the principal investigator (ML).

## Declaration of competing interest

Corinne Levy reports travel grants from Pfizer and personal fees from Pfizer and MSD. The other authors have no conflicts of interest to disclose.

The authors declare the following financial interests/personal relationships which may be considered as potential competing interests:

Michael Levy reports financial support was provided by AXA International Assurance. If there are other authors, they declare that they have no known competing financial interests or personal relationships that could have appeared to influence the work reported in this paper.

## Annex 1 CAPRICE Study Group

Julien Baleine, Laurent Balu, Simon Barreault, Clémence Beauruelle, Emma Becuwe Gosselin, Matthieu Bendavid, Xavier Beretta Piccoli, Lionel Berthomieu, Stéphane Blanot, Stephane Bonacorsi, Yvonnick Boué, Noel Boussard, Gérald Boussicault, Camille Brehin, Alexandre Bretonneau, Arthur Charazac, Camille Chavy, Maryline Chomton Cailliez, Benoit Colomb, Fleur Cour Andlauer, Julie Cremninter, Etienne Darieux, Stephane Dauger, Alexandra David, Sandrine De Sampaio, Damien Dubois, Audrey Dupont, Jean Duval Destin, Aben Essid, Marion Favre, Fiona Bensoussan, Ghida Ghostine, Lucie Gibold, Marie-Laure Girardin, Chloe Hild, Sylvia Iacobelli, Charlotte Idier, Christophe Isnard, Benoit Jaulhac, Katy Jeannot, Julien Jegard, Mikael Jokic, Aurélie Labarre, Elise Launay, Cécile Le Brun, Michael Levy, Alain Lozniewski, Annelise Mary, Fabrice Michel, Christophe Milesi, Julie Missaire, Guillaume Mortamet, Simon Mounier, Audrey Mowendabeka, Jérôme Naudin, Anouk Navion, Mehdi Oualha, Naim Ouldali, Bruno Ozanne, Carla Parada-Rodrigues, Manon Passard, Quentin Pledran, Amandine Prenant, Pauline Ragon, Jérôme Rambaud, Sophie Reissier, Chloé Ribet, Claire Richarme, Barbara Ros, Jean-Michel Roué, Nicolas Roullet-Renoleau, Jérémie Rousseaux, Montserrat Sierra Colomina, Laurence Tabone, Muhamed-Kheir Taha, Asmaa Tazi, Aurore Thollot, Nicolas Traversier, Anne Tristan, Lucile Tzaroukian, Noemie Vanel, Diane Vareilles, Emmanuelle Varon, Laurent Villeneuve, Evelyne Werner, Arnaud Wiedemann, Amani Zaouali Dridi, Justine Zini, Charlie Zins
