# Supplementary material for: A contemporary overview of severe community-acquired bacterial infections in pediatric intensive care units
Source: Ann Intensive Care. 2026 Jul 9;16:100112. doi: 10.1016/j.aicoj.2026.100112 (PMC13396938; doi:10.1016/j.aicoj.2026.100112)
Supplement: Supplementary file 1 [file mmc1.docx]

**A contemporary overview of severe community-acquired bacterial infections in paediatric intensive care units**

**Supplementary Appendix**

**eTable 1. Diagnosis methods for each pathogen included in the study**

| **Pathogen** | **Methods used** |
| --- | --- |
| *Bordetella pertussis* | Nucleic acid amplification test |
| *Mycoplasma pneumoniae* | Nucleic acid amplification test |
| *Streptococcus pneumoniae* | Culture or nucleic acid amplification test or rapid antigene detection test |
| *Staphylococcus aureus* | Culture or nucleic acid amplification test |
| *Escherichia coli* | Culture or nucleic acid amplification test |
| *Group A streptococcus* | Culture or nucleic acid amplification test or rapid antigene detection test |
| *Neisseria meningitidis* | Culture or nucleic acid amplification test |
| *Haemophilus influenzae* | Culture or nucleic acid amplification test |
| *Group B streptococcus* | Culture or nucleic acid amplification test |
| *Oral streptococcus* | Culture or nucleic acid amplification test |
| *Salmonella* | Culture or nucleic acid amplification test |
| *Fusobacterium necrophorum* | Culture or nucleic acid amplification test |
| *Klebsiella* | Culture or nucleic acid amplification test |
| Other pathogenes | Culture or nucleic acid amplification test |

**eTable 2: Demographic characteristics and evolution according to age category in patients with a community-acquired bacterial infection admitted to a pediatric intensive care unit in 2024.**

|  |  | **Age group, n (%)** | | | | | |  |
| --- | --- | --- | --- | --- | --- | --- | --- | --- |
|  | **Total** | **<3 months (n=252)** | **3–11 months (n=134)** | **12 months–5 years**  **(n=176)** | **5–12 years**  **(n=194)** | **12–18 years**  **(n=141)** | | |
| **Male sex** | 470 | 128 (51%) | 80 (60%) | 94 (53%) | 96 (49%) | 72 (51%) |  |  |
| **Chronic condition** |  |  |  |  |  |  |  |  |
| Chronic condition | 265 | 35 (14%) | 34 (25%) | 69 (39%) | 69 (36%) | 58 (41%) |  |  |
| Immune deficit | 30 | 1 (0.1%) | 1 (0.5%) | 6 (3%) | 14 (5%) | 8 (6%) |  |  |
| **Clinical presentation** |  |  |  |  |  |  |  |  |
| Diffuse pneumonia | 200 | 86 (34%) | 18 (13%) | 33 (19%) | 42 (22%) | 21 (15%) |  |  |
| Pleuropneumonia | 102 | 18 (8%) | 13 (10%) | 19 (11%) | 25 (13%) | 27 (19%) |  |  |
| Lobar pneumonia | 84 | 19 (8%) | 6 (4%) | 19 (11%) | 23 (12%) | 17 (12%) |  |  |
| Meningitis | 175 | 44 (18%) | 46 (34%) | 40 (23%) | 26 (13%) | 19 (13%) |  |  |
| Ear-nose-throat infection | 118 | 10 (4%) | 27 (20%) | 33 (19%) | 32 (16%) | 16 (11%) |  |  |
| Abdominal infection | 46 | 6 (2%) | 3 (2%) | 10 (6%) | 17 (9%) | 10 (7%) |  |  |
| Cerebral empyema | 46 | 4 (2%) | 3 (2%) | 9 (5%) | 15 (8%) | 15 (11%) |  |  |
| Urinary tract infection | 43 | 17 (7%) | 8 (6%) | 4 (2%) | 3 (2%) | 11 (8%) |  |  |
| Soft-tissue infection | 36 | 5 (2%) | 7 (5%) | 6 (3%) | 9 (5%) | 9 (6%) |  |  |
| Infection without a source | 33 | 4 (2%) | 6 (4%) | 6 (3%) | 11 (6%) | 6 (4%) |  |  |
| Osteoarticular infection | 14 | 3 (1%) | 3 (2%) | 0 (0%) | 4 (2%) | 4 (3%) |  |  |
| Other | 104 | 9 (4%) | 16 (12%) | 27 (15%) | 29 (17%) | 23 (16%) |  |  |
| **Sepsis and septic shock** |  |  |  |  |  |  |  |  |
| Sepsis | 114 | 18 (7%) | 28 (21%) | 28 (16%) | 21 (11%) | 19 (13%) |  |  |
| Septic shock | 149 | 34 (13%) | 20 (15%) | 27 (15%) | 32 (16%) | 36 (26%) |  |  |
| **Number of organ failures** |  |  |  |  |  |  |  |  |
| 1 | 230 | 57 (23%) | 26 (19%) | 50 (28%) | 68 (35%) | 29 (21%) |  |  |
| ≥2 | 275 | 72 (29%) | 51 (38%) | 45 (26%) | 55 (28%) | 52 (37%) |  |  |
| **Outcome** |  |  |  |  |  |  |  |  |
| Mortality at day 28 | 60 | 27 (11%) | 8 (6%) | 9 (5%) | 8 (4%) | 8 (6%) |  |  |
| Sequelae at day 28 | 126 | 21 (8%) | 21 (16%) | 22 (13%) | 39 (20%) | 23 (16%) |  |  |

**eTable 3. Comparison of patients admitted to the pediatric intensive care unit for community-acquired bacterial infections according to the presence or absence of sepsis or septic shock**

|  | **Sepsis or septic shock* (n=263)** | **No sepsis or septic shock (n=618)** | **p-value** |
| --- | --- | --- | --- |
| **Age** | 2.5 (0.4 ; 10.7) | 1.7 (0.2 ; 8.1) | 0.011 |
| **Sex** |  |  |  |
| Male sex | 147 (56%) | 322 (52%) | 0.331 |
| **Chronic condition** | 83 (32%) | 181 (29%) | 0.705 |
| **Clinical presentation** |  |  |  |
| Diffuse pneumonia | 21 (8%) | 179 (29%) | <0.001 |
| Pleuropneumonia | 36 (14%) | 66 (11%) | 0.172 |
| Lobar pneumonia | 20 (8%) | 62 (10%) | 0.288 |
| Meningitis | 72 (27%) | 102 (17%) | <0.001 |
| Ear-nose-throat infection | 29 (11%) | 89 (14%) | 0.209 |
| Abdominal infection | 25 (10%) | 21 (3%) | <0.001 |
| Cerebral empyema | 11 (4%) | 35 (4%) | 0.395 |
| Urinary tract infection | 18 (7%) | 25 (4%) | 0.068 |
| Soft-tissue infection | 19 (7%) | 17 (3%) | 0.002 |
| Infection without a source | 33 (263) | 0 (0%) | <0.001 |
| Osteoarticular infection | 5 (2%) | 9 (2%) | 0.475 |
| Other infection | 61 (23%) | 43 (7%) | <0.001 |
| **Delay 1^st^ symptom to PICU admission, days** | 2 (1 ; 4) | 4 (2 ; 7) | 0.005 |
| **Pathogens** |  |  |  |
| *Streptococcus pneumoniae* | 36 (14%) | 65 (11%) | 0.149 |
| *Neisseria meningitidis* | 32 (12%) | 21 (3%) | <0.001 |
| *Group A streptococcus* | 21 (8%) | 40 (6%) | 0.380 |
| *Group B streptococcus* | 13 (5%) | 15 (2%) | 0.046 |
| *Haemophilus influenzae* | 13 (5%) | 39 (6%) | 0.465 |
| *Escherichia coli* | 18 (7%) | 44 (17%) | 0.936 |
| *Staphylococcus aureus* | 42 (16%) | 43 (7%) | <0.001 |
| *Bordetella pertussis* | 10 (4%) | 167 (27%) | <0.001 |
| *Mycoplasma pneumoniae* | 11 (4%) | 108 (17%) | <0.001 |
| Other bacteria | 47 (18%) | 77 (12%) | 0.027 |
| **Viral coinfection** | 79 (30%) | 223 (36%) | 0.361 |

*^*^according to the Phoenix sepsis score*

*PICU : pediatric intensive care unit*
