# Supplementary material for: A contemporary overview of severe community-acquired bacterial infections in pediatric intensive care units
Source: Ann Intensive Care. 2026 Jul 9;16:100112. doi: 10.1016/j.aicoj.2026.100112 (PMC13396938; doi:10.1016/j.aicoj.2026.100112)
Supplement: Supplementary file 2 [file mmc2.docx]

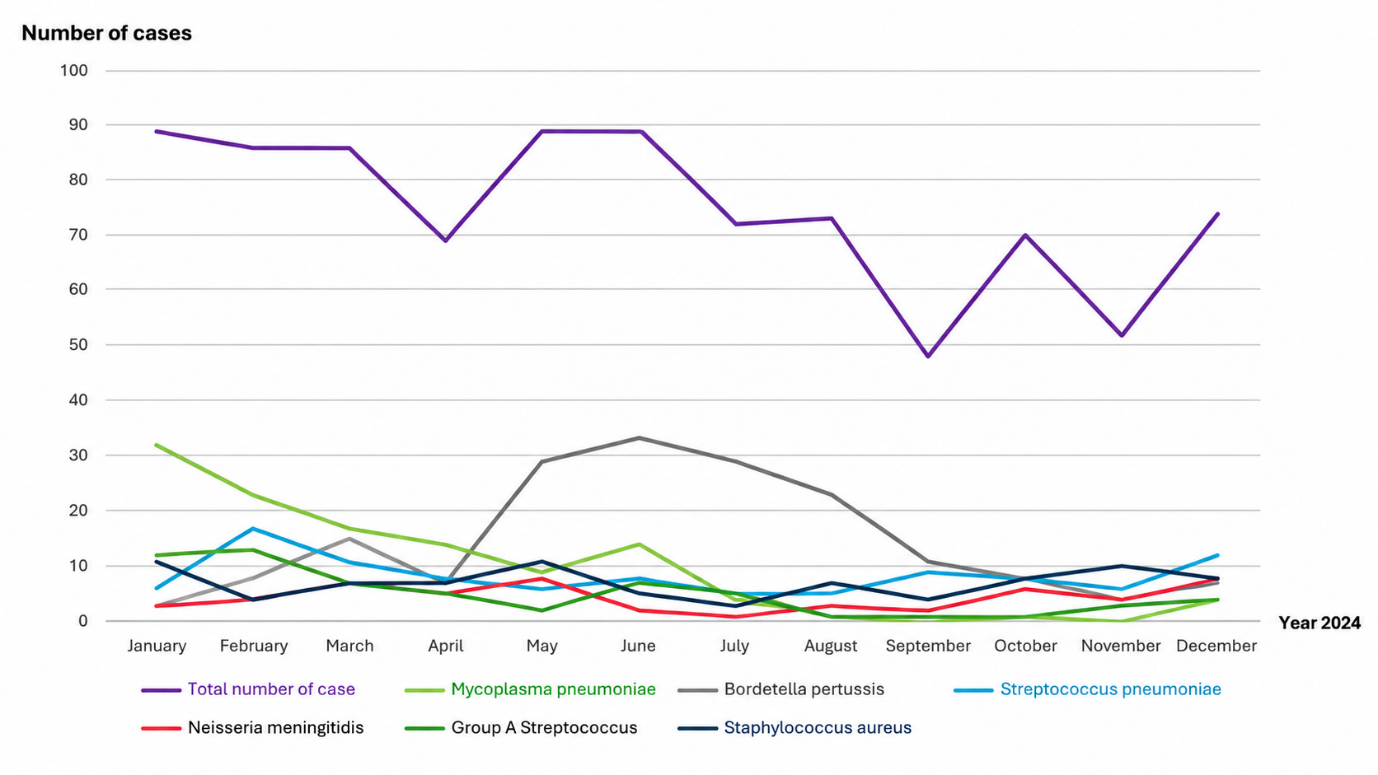


**Supplemental Figure 1**. Monthly Incidence of the main invasive pathogens of community-acquired bacterial infection in children admitted to a French pediatric intensive care unit in 2024.
